# Predictors of mosquito net use in Ghana

**DOI:** 10.1186/1475-2875-10-265

**Published:** 2011-09-15

**Authors:** Carol A Baume, Ana Cláudia Franca-Koh

**Keywords:** ITN, LLIN, mosquito net, bednet, net use, free nets, private sector, market, coils

## Abstract

**Background:**

During the past decade the malaria control community has been successful in dramatically increasing the number of households that own mosquito nets. However, as many as half of nets already in households go unused. This study examines the factors associated with use of nets owned in Ghana.

**Methods:**

The data come from an August 2008 survey in Ghana of households with a pregnant woman or a guardian of a child under five, conducted during the rainy season. 1796 households were included in this analysis, which generated a sample of 1,852 mosquito nets. Using each net owned as the unit of analysis, multivariate logistic regression was used to examine the relationship of *net used last night *with 23 potentially explanatory variables having to do with characteristics of the household, of the respondent, and of the net. Odds Ratios, p-values, and confidence intervals were calculated for each variable to develop an explanatory model.

**Results:**

The final multivariate model consisted of 10 variables statistically associated with whether or not the net was used the prior night: rural location, lower SES, not using coils for mosquito control, fewer nets in the household, newer nets and those in better condition, light blue colour, higher level of education of the guardian of the child under five, knowing that mosquitoes transmit malaria, and paying for the net instead of obtaining it free of charge.

**Conclusions:**

The results of this study suggest that net use would increase in Ghana if coloured nets were made available in mass distributions as well as in the commercial market; if programmes emphasize that malaria is caused only by night-biting mosquitoes, and that nets protect against mosquitoes better than coils and need to be used even if coils are burning; if donated nets are replaced more frequently so that households have nets that are in good condition; and if there were support for the commercial market so that those who can afford to purchase a net and want to choose their own nets can do so.

## Background

During the past decade, the malaria control community has been successful in dramatically increasing the number of households that own mosquito nets – particularly ITNs (insecticide-treated nets) and LLINs (long-lasting insecticide-treated nets) – via programmes that made nets available commercially, at subsidized prices, and free of charge to families via mass distributions. However, as many as half of nets owned by households go unused [1-3], and there are few systematic studies to understand why a family would not use a net already present in the house. Most studies of net use attempt to explain why vulnerable groups such as children under five or pregnant women are or are not under a net [4-10] or describe which household members use the household's net(s) [11-13]. Few studies seeking to document and understand use and non-use of nets owned look at all the nets in the sample to measure the proportion used the prior night and analyse the factors associated with their use, including characteristics of the net itself.

The present study analyses the factors related to use of mosquito nets – including ITNs, LLINs, and untreated nets – owned in Ghana. Understanding why a net already in the household is used or not is essential for malaria control efforts. No matter how effective a mosquito net is in the laboratory, it cannot provide optimal protection unless it is used.

## Methods

### Study approach

The data come from a household survey on mosquito nets conducted in Ghana in August 2008 during the rainy season, as part of systematic monitoring carried out by the USAID-funded NetMark Project at AED [14]. The survey included an enumeration of each net in the household and information about size, shape, colour, source, cost, treatment status (ITN/LLIN or not), and whether or not it had been slept under the prior night. In this article, the term "net" refers to any mosquito net owned by the household, including ITNs, LLINs, and non-ITNs; all are included in order to use treatment status as an independent variable. The exception is baby nets – small, non-hanging nets with a built-in frame – which have been excluded from enumeration since families get them for infants and keep them (unused) even when the child grows out of it. In addition to questions on ownership and use, the survey included questions on knowledge and beliefs about mosquitoes and malaria; perceptions of treated and untreated mosquito nets, use of other mosquito control products, and consumer preferences regarding shape, colour, and size of nets.

The survey also included questions on ownership of assets, household characteristics, and level of education in order to develop a scale for socioeconomic status (SES). Most of the variables were drawn from the Demographic and Health Surveys. Principal components analysis was used to derive factor scores, and households were divided into quintiles based on their factor scores.

### Sample

The sample consisted of 1,796 households from six of the ten regions in Ghana: Accra, Ashanti, Central, Volta, Northern, and Upper West. Using Ghana's census data from the year 2000, 180 localities were randomly selected, with the number of localities selected per region based on probability proportionate to the region's population size. Ten households per locality were selected for interview by having the field team disperse in different directions within the locality and screen for eligibility. Eligible respondents were women of reproductive age (15-49 years old) who were pregnant or mothers/guardians of a child under five years of age. Only one respondent per household was interviewed. A total of 1,800 interviews were conducted, but four households were dropped from the dataset for insufficient data.

### Data management and analysis

Responses to the survey were entered directly into PDAs (personal digital assistants, or handheld computers). The PDAs were programmed with built-in skip patterns and data checks so that it was not possible to enter an out-of-range response or to skip a question. The resulting data was converted to an SPSS database with each household/respondent as a case (household-level file). The data were restructured into a separate file with each net owned as the unit of analysis (net-level file). The net-level file was used for this analysis of net use: using each net owned as the unit of analysis, multivariate logistic regression was used to examine the relationship of the dependent variable "*net used or not last night" *with 23 potentially explanatory independent variables having to do with characteristics of the household, of the respondent, and of the net, specifically:

#### Household background characteristics

Urban or rural location; region; household head years of education; family size; number of children under five years of age; number of pregnant women; number of nets in household; use of coils for mosquito control; whether or not household received indoor residual spraying (IRS) within the past year, and socioeconomic status (SES).

#### Respondent characteristics

Years of education; knowledge that mosquitoes transmit malaria.

#### Characteristics of the net

Whether or not net is currently treated (i.e.is an LLIN or ITN); age, size, colour, and shape of net; condition of net in terms of wear; cleanliness of net; where net was obtained; brand of net; whether net was free or purchased.

Given a dichotomous dependent variable and multiple independent variables, multivariate logistic regression was selected as the appropriate analytic procedure. The analysis was conducted in STATA. A multivariate logistic regression model was built using the process recommended by Hosmer & Lemeshow [15] known as "purposeful selection," as follows:

(1) First, the relationship of each of the independent variables with the dependent variable is investigated. Each is tested by fitting univariate logistic regression models and estimating odds ratios (OR). Those variables having a p-value of less than 0.25 are retained.

(2) For the variables retained, a correlation analysis is conducted to test for collinearity. Where correlations occur at 0.6 or higher, only one of the variables is kept for inclusion in the multivariate model. The variable to be included was chosen by examining the strength of the relationship with the dependent variable. The variable having a smaller p-value-and therefore greater statistical significance-is retained, and the other(s) removed.

(3) Once all independent variables significant at p < 0.25 are identified and correlations checked, the remaining variables are entered together in a multivariate model. Those not significant at p < 0.05 level are then removed one by one, resulting in a smaller model.

(4) After the smaller model is reached-where all the independent variables were significant at p < 0.05-each variable that had been removed from the initial model is checked to make sure it was still non-significant. If a variable is significant after it is re-entered into the smaller model, it is kept in the model. These checks are conducted until it is clear that the best final multiviariate model with all variables significant is obtained.

## Results

### Descriptive

The majority of the 1796 households surveyed-71%-owned at least one net, with an average of 1.5 nets per net-owning household. The total number of nets owned by households in the sample was 1852, which constituted the sample for this analysis. Among those, 76% were LLINs, 10% were ITNs but not LLINs, and 14% were non-ITNs (i.e., were untreated or the treatment had expired). Most nets (83%) were double-sized; 8% were triple; 8% single, and less than 1% cot or crib sized. The great majority of nets (95%) were rectangular in shape; almost all of the rest were conical. The most common colour was white (63%), followed by light blue (18%), dark blue (7%), and green (7%). In terms of age, 43% of nets were less than a year old, 18% were at least 1 year old but less than two years old, 25% were at least two years old but less than three years old, 8% were at least three years old but less than four years old, and 5% were four or more years old.

Nets were classified as free, purchased, or other. Purchased nets included those bought at subsidized prices, those bought with vouchers, and those bought at full commercial prices. A majority (64%) of nets owned had been acquired free of charge; 34% had been purchased; and 2% were acquired as a gift or through trade or barter.

Of nets owned, 59% reportedly had been used the prior night-i.e., had someone sleeping under them the night before the survey. About 1/5 (21%) of nets owned were still in the sealed package, thus had never been used.

### Univariate analysis

Of the 23 independent variables analysed, 16 were found to be associated with net use at p < 0.25 and were retained for further analysis. (See Additional File 1). At the household level, those were region (Volta and upper West), rural location, lower SES, higher levels of education for household head, not using coils for mosquito control in the prior year, and fewer nets in the household. At the respondent level, both of the variables – higher years of education and knowing that mosquitoes transmit malaria – were associated with net use. Of net characteristics, those associated with the net being used the prior night at p < 0.25 were newer age of net; colour (light blue, green), size (double), condition of net (worn with and without holes); cleanliness of net (only slightly dirty); source of net (obtained from a drug store or pharmacy); brand of net (PermaNet^®^); and having been purchased rather than obtained free of charge.

After determining which independent variables were related to the net being used the prior night at criterion level p < 0.25, associations between these variables were checked to avoid collinearity. Only one pair of variables was significantly associated: condition of net (in terms of wear/holes) and cleanliness of net (Chi-square p = 0.000). Condition of net was retained, based on its stronger relationship with the dependent variable, and cleanliness of net was dropped.

### Multivariate analysis

The final multivariate model consisted of 10 variables that had an association with a net being used the prior night at a significance level of p < 0.05. (See Table 1.) In this final model, a net was significantly more likely to be used if the household it belonged to was in a rural rather than urban area (OR = 1.92 [95% C.I. 1.49-2.47]; p = 0.000); was of lower SES status (OR = 1.11 for each level decrease in SES [95% C.I. 1.00-1.22]; p < 0.05); had not used coils for mosquito control in the prior 12 months (OR = 1.48 [95% C.I. 1.20-1.83]; p = 0.000), and had fewer nets – no more than one (OR = 7.47 [95% C.I. 4.87-11.43]; p = 0.000), or two (OR = 1.93 [95% C.I. 1.23-3.02]; p < 0.01) compared to three or more nets.

**Table 1:** Final Multivariate Logistic Regression Model: Predictors of "Net used last night"

INDEPENDENT VARIABLES	OR^	P-VALUE^	95% CI^
**Urban-Rural**			

Urban	1.0		

Rural***	1.92	0.000	1.49-2.47

**SES**			

Highest level of SES	1.0		

Each level decrease in SES*	1.11	0.045	1.00-1.22

**Used coils in past 12 months**			

Yes	1.0		

No***	1.48	0.000	1.20-1.83

**Number of nets in household**			

3 or more nets	1.0		

Two nets**	1.93	0.004	1.23-3.02

One net***	7.47	0.000	4.87-11.43

**Free net/purchased net**			

Free	1.0		

Purchased***	1.88	0.000	1.49-2.38

Other (gift/trade/barter)	1.0	0.996	0.48-2.10

**Age of net**			

4 or more years	1.0		

3 years	1.62	0.109	0.90-2.91

2 years**	2.30	0.002	1.37-3.86

1 year old**	2.39	0.001	1.40-4.07

< 1 year old**	2.44	0.001	1.46-4.05

**Condition of net**			

New, like new	1.0		

Worn, no holes***	5.60	0.000	3.65-8.57

Worn, holes***	3.12	0.000	1.67-5.84

Did not see net***	2.05	0.000	1.60-2.61

**Colour of net**			

White	1.0		

Light blue*	1.38	0.024	1.04-1.83

Dark blue	0.82	0.353	0.55-1.24

Turquoise	1.12	0.741	0.58-2.17

Green	1.40	0.118	0.92-2.12

Other	0.87	0.739	0.38-1.97

**Respondent years of education**			

None	1		

1-6 years**	1.66	0.006	1.15-2.38

7-9 years*	1.48	0.016	1.08-2.03

10-12 years***	2.32	0.000	1.58-3.40

13 or more years***	2.32	0.001	1.41-3.83

**Knows that mosquitoes transmit malaria**			

No	1		

Yes*	1.38	0.033	1.03-1.86

N = 1852

^OR, adjusted odds ratio; P-value for Wald statistic; CI, 95% confidence interval

*p < 0.05, **p < 0.01, ***p < 0.001

Pseudo R2: 0.1521

At the respondent level, nets owned by respondents who were of higher educational level (for example, 10 to 12 years of education compared to none (OR = 2.32 [95% C.I. 1.58-3.40]; p = 0.000); and knew that mosquitoes transmit malaria (OR = 1.38 [95% C.I. 1.03-1.86]; p < 0.05), were also significantly more likely to have been used the prior night.

In terms of net characteristics, a net was significantly more likely to have been used the prior night if it was newer in age (for example, less than a year old compared to 4 or more years old (OR = 2.44 [95% C.I. 1.46-4.05]; p < 0.01); showed signs of wear compared to new (OR = 5.60 [95% C.I. 3.65-8.57]; p = 0.000), including holes (OR = 3.12 [95% C.I. 1.67-5.84]; p = 0.000); was light blue as opposed to white (OR = 1.38 [95% C.I. 1.04-1.83]; p < 0.05); and had been paid for rather than obtained free of charge (OR = 1.88 [95% C.I. 1.49-2.38]; p = 0.000).

## Discussion

Over 40% of nets in this sample had not been used the prior night – a large proportion that represents considerable loss of protection against malaria, especially since the survey took place during the rainy season. The intent of this analysis was to shed light on the factors that affected whether a net owned by a household was used the previous night, using the net as the unit of analysis. The final multivariate model from this study found that the factors associated with whether a given net was used the prior night were rural location, lower SES, not using coils for mosquito control, fewer nets in the household, newer nets and those in better condition, light blue colour, higher level of education of the mother, knowing that mosquitoes transmit malaria, and paying for the net instead of obtaining it free of charge.

Some of these findings confirm intuitive reasoning. For example, old nets were less likely to be used than newer ones; the data show that a net that is less than a year old is far more likely to be used than a net that is four or more years old. Old nets are likely to be worn and unattractive, and possibly ineffective. Similarly, a net that showed signs of wear but had no holes was far more likely to be in use than one that was "new/like new"-since most nets categorized as "new/like new" were still in the package, unused. Nets with holes were also more likely than a "new/like new" net to be used-not as likely as one without holes but more likely than one still in package or kept carefully stored. The worn condition is the effect of use rather than something that prompts use, but when a net gets so worn that it has large holes, it discourages use since it provides less protection against malaria and nuisance biting.

The mother/guardian's educational level and knowledge were found to be associated with a net being used, as in other studies [1,10,16-18]. This analysis found that the odds of a net being used in a household where the mother/guardian had at least 10 years of education were 2.32 compared to a net owned by a respondent with no education. The odds of a net being used belonging to a mother/guardian who knew that mosquitoes transmit malaria was 1.4 in comparison to a net belonging to a mother who did not know the transmission vector.

The odds of a particular net being used decreased with the number of nets owned by the household. In other words, the more nets available in the household, the less likely that a given net will be used. Indeed, 21% of nets owned had never been used and were still in the package. Research in Ethiopia on unused nets found that households may keep additional net(s) unused because there is no room to hang it, or keep it in reserve net for when the one being used wears out, or keep it as an asset that can be converted to cash when needed. Families that were well off might keep an extra net for guests [19].

The finding that a net is more likely to be used if there are fewer nets in the household may appear to contradict other studies that find that a child under five or a pregnant women is more likely to sleep under a net if there are *more *nets in the household [4,5,10]. However, those studies have a slightly different focus, which is to look at the factors associated with a child or pregnant woman using a net, since a Roll Back Malaria indicator is the percent of vulnerable groups sleeping under a net; therefore in those studies the child or pregnant woman is the unit of analysis. This study uses the net as the unit of analysis since it is focused on use and non-use of nets in the household, which is of particular interest in the wake of large-scale bednet distributions that intend to put multiple nets in households, allow bednet use beyond vulnerable groups, and extend some protection even to those not sleeping under a net by killing and repelling more mosquitoes. The two findings are not contradictory; they result from examining different questions. It is important to distinguish between the two, especially when making policy decisions.

In this analysis, 63% of nets owned were white but light blue nets were more likely to be used than white nets. Light blue nets are available in the commercial market in Ghana and were also distributed in some areas free of charge to the household. Possibly light blue is preferred to white because colour does not show dirt as readily as white, or because a coloured net is considered decorative. Although colour of net was statistically significant, shape and size of net, as well as brand, were not. Brand did just border significance in the univariate analysis, with Olyset^® ^less likely to be used than PermaNet^®^-but dropped out when adjusted for the other variables in the final model. Shape of net was a strong predictor of use in Ethiopia as well as Sri Lanka [19,20], with conical nets much more likely to be in use than rectangular ones. Conical nets fit rural houses in Ethiopia more readily than rectangular nets do, but in Ghana that may not be the case.

Surprisingly, treatment status of net-untreated versus ITN/LLIN-did not show an association with use. The great majority of nets were LLINs, but it might be expected that the small proportion of untreated nets (10%) would be less likely to be in use.

The odds of a net from a household that had used coils for mosquito protection in the prior year being used were lower than for a net from a household that had not used coils, suggesting that households use coils to substitute for net use. The survey from which the data were drawn found that coils were used across the socio-economic spectrum among 58% of households, and that about 43% of those households used them virtually daily. In Ghana, it may be important to dispel the idea that nets are not needed if you burn coils.

Although use of coils appeared to substitute for net use, use of commercial aerosol spray and having one's house sprayed with IRS did. Perhaps coils, because of their low price, can be used consistently whereas aerosol spray cannot; only 18% of the lowest SES households had used aerosol spray in the prior year, compared with 63% of the highest SES households. It is also probable that aerosols are not particularly effective in rural and poor urban homes where it is difficult to close off rooms. One might surmise that nets would be less likely to be used in households receiving indoor residual spraying, as was found in a study in Mozambique and Nigeria [21,22]. The lack of statistical relationship with IRS in this study may have to do with the small number of households in the sample that had been sprayed in the prior year: 104, or 5.6% of the sample.

A net that was purchased was much more likely to be used than one acquired free of charge. The net use study in Ethiopia that also used the net as the unit of analysis found the same result [19]. An item that is purchased is one that is wanted or needed. If a decision is made to purchase a net, it implies that the benefits of having-and using-a net are recognized, which is not necessarily the case if the net has been given. Further, if a net is purchased, the desired size and colour can be selected. The finding suggests that it is important to devise strategies for supporting the commercial market for nets while making nets available through mass distribution. Such a strategy would also lay the foundation for a sustained supply of nets.

There are some limitations of this study. The data collection instrument was not designed specifically for a net use study; secondary analysis was conducted to look at the issue. A study designed specifically for net use would ideally include attitudinal questions about perceived mosquito risk at the time of interview (since mosquito population levels vary by season and by locale) and perceived current effectiveness of the net in protecting against mosquitoes; size of the house in terms of space for hanging nets and use of space in terms of dedicated to sleeping versus multiple use; and shape of the house to look at ease or difficulty of hanging different types of nets within the house structure. All of these have been found to influence whether or not nets already present in the household are used [19,23,24].

## Conclusions

There is concerted work by the LLIN community to increase use of nets owned to maximize their impact on reducing the burden of malaria. The results of this study suggest that net use would increase in Ghana if coloured nets were made available in mass distributions as well as in the commercial market; if programmes emphasize that malaria is caused only by night-biting mosquitoes, and that nets protect against malaria better than coils and need to be used even if coils are burning; if donated nets are replaced more frequently so that households have nets that are in good condition; and if there were support for the commercial market so that those who can afford to purchase a net and want to choose their own nets can do so.

## Competing interests

The authors declare that they have no competing interests.

## Authors' contributions

CAB designed and implemented the survey and wrote the article. AFK conducted the data analysis. Both authors read and approved the final manuscript.

## Authors' information

CAB was Research Director for the NetMark project at AED and AFK was on the research staff. CAB is currently an independent consultant and AFK is a researcher at ICF International.

## Supplementary Material

Additional file 1 **Supplementary table: Univariate Logistic Regression Results for Predictors of "Net used last night"**. Click here for file
