# Supplementary material for: Predictors of mosquito net use in Ghana
Source: Malar J. 2011 Sep 15;10:265. doi: 10.1186/1475-2875-10-265 (PMC3196744; doi:10.1186/1475-2875-10-265)
Supplement: Additional file 1 — Supplementary table: Univariate Logistic Regression Results for Predictors of "Net used last night". [file 1475-2875-10-265-S1.DOCX]

**Additional File 1**

**Univariate Logistic Regression Results for Predictors of “Net used last night”**

| **I N D E P E N D E N T**  **V A R I A B L E S** | **N** | **%** | **OR^** | **P-VALUE^** | **95% CI^** |
| --- | --- | --- | --- | --- | --- |
| **HOUSEHOLD CHARACTERISTICS** | | | | | |
| **Urban-Rural***  Urban  Rural | 910  942 | 49.1  50.9 | 1.0  1.71 | 0.000 | 1.42-2.07 |
| **Region***  Accra/Eastern  Ashanti  Central  Volta  Northern  Upper West | 621  510  265  170  189  97 | 33.5  27.5  14.3  9.2  10.2  5.2 | 1.0  1.03  1.08  1.60  1.00  1.55 | 0.795  0.619  0.010  0.987  0.057 | 0.81-1.31  0.80-1.44  1.12-2.29  0.72-1.39  0.99-2.44 |
| **SES***  1 Low  2  3  4  5 High  ***********************  Highest level of SES  Each level decrease in SES | 342  378  331  399  402 | 18.5  20.4  17.9  21.5  21.7 | 1.0  1.13 | 0.000 | 1.06-1.21 |
| **Household Head Years of Education***  None  1-6 years  7-9 years  10-12 years  13 or more years  Don’t know or missing | 240  94  390  513  374  241 | 13.0  5.1  21.1  27.7  20.2  13.0 | 1.0  1.33  1.00  1.31  1.24  0.90 | 0.249  0.987  0.088  0.208  0.549 | 0.82-2.18  0.73-1.39  0.96-1.79  0.89-1.72  0.63-1.28 |
| **Family size**  1-2  3  4  5  6  7  8  9  10-14  ***********************  First level  Each level increase in family size | 110  390  545  422  193  83  45  44  20 | 5.9  21.1  29.4  22.8  10.4  4.5  2.4  2.4  1.1 | 1.0  1.01 | 0.680 | 0.96-1.07 |
| **# of children <5 years**  0  1  2  3  4-5  ***********************  First level  Each level increase in # of children <5 | 120  1130  498  85  19 | 6.5  61.0  26.9  4.6  1.0 | 1.0  0.98 | 0.735 | 0.86-1.11 |
| **# of pregnant women**  None  One | 1566  286 | 84.6  15.4 | 1.0  0.97 | 0.808 | 0.75-1.25 |

| **Used coils in past 12 months***  Yes  No | 917  935 | 49.5  50.5 | 1.0  1.20 | | 0.056 | | 1.00-1.44 |  |
| --- | --- | --- | --- | --- | --- | --- | --- | --- |
| **Used aerosol insecticide spray in past 12 months**  No  Yes | 1235  617 | 66.7  33.3 | 1.0  1.02 | | 0.852 | | 0.84-1.24 |  |
| **Walls were sprayed (IRS) in past 12 months**  No  Yes | 1748  104 | 94.4  5.6 | 1.0  1.16 | | 0.302 | | 0.55-1.21 |  |
| **Number of nets***  3 or more nets  Two nets  One net | 136  451  1265 | 7.3  24.4  68.3 | 1.0  1.92  6.40 | | 0.003  0.000 | | 1.25-2.94  4.28-9.58 |  |
| **MOSQUITO NET CHARACTERISTICS** | | | | | | | |  |
| **Free/paid***  Free  Paid  Other (gift/trade/barter) | 1185  629  38 | 64.0  34.0  2.1 | 1.0  1.54  0.90 | | 0.000  0.747 | | 1.26-1.88  0.47-1.72 |  |
| **Age of net***  4 or more years  3 years  2 years**  1 year old**  < 1 year old** | 92  149  455  364  792 | 5.0  8.05  24.6  19.7  42.8 | 1.0  1.34  1.75  1.63  1.38 | | 0.260  0.015  0.037  0.143 | | 0.80-2.27  1.11-2.75  1.03-2.59  0.90-2.13 |  |
| **Shape of net**  Rectangular  Round/Conical  Triangle/Pyramid  Wedge | 1756  84  6  6 | 94.8  4.5  0.3  0.3 | 1.0  0.98  0.70  1.41 | | 0.945  0.667  0.694 | | 0.63-1.53  0.14-3.49  0.26-7.70 |  |
| **Size of net***  Triple/King  Double  Single  Cot/Crib | 150  1544  151  7 | 8.1  83.4  8.2  0.4 | 1.0  1.42  1.61  0.73 | | 0.044  0.043  0.687 | | 1.01-1.98  1.01-2.54  0.16-3.38 |  |
| **Condition of net***  New, like new  Worn, no holes  Worn, holes  Did not see net | 494  219  63  1076 | 26.7  11.8  3.4  58.1 | 1.0  4.94  2.12  1.65 | | 0.000  0.007  0.000 | | 3.37-7.75  1.23-3.67  1.33-2.05 |  |
| **Colour of net***  White  Light blue  Dark blue  Turquoise  Green  Other | 1176  332  131  49  134  30 | 63.5  17.9  7.1  2.7  7.2  1.6 | 1.0  1.26  0.80  1.07  1.37  1.11 | | 0.074  0.222  0.813  0.099  0.783 | | 0.98-1.62  0.56-1.15  0.60-1.92  0.94-1.99  0.53-2.32 |  |
| **Net is ITN/LLIN**  No  Yes | 253  1599 | 13.7  86.3 | 1.0  1.15 | | 0.303 | | 0.89-1.50 |  |
| **Cleanliness of net***  Very clean  A bit dirty  Very dirty  Did not see net | 485  253  37  1077 | 26.2  13.7  2.0  58.2 | 1.0  4.89  1.09  1.67 | | 0.000  0.810  0.000 | | 3.41-7.03  0.56-2.12  1.34-2.07 |  |
| **Source where net was obtained***  Health Facility  Child Health or Immunization Day  Unspecified retail outlet, using voucher  Open air/Structured market  Gift  Chemical store/Drug shop  Other  Don’t know | 894  302  242  104  62  54  176  17 | 48.3  16.3  13.1  5.6  3.4  2.9  9.5  0.9 | 1.0  1.03  0.61  1.07  0.76  1.74  1.01  0.95 | | 0.844  0.001  0.755  0.298  0.076  0.946  0.925 | | 0.79-1.34  0.46-0.82  0.70-1.62  0.45-1.27  0.94-3.20  0.73-1.41  0.36-2.53 |  |
| **Brand of net***  Permanet  Olyset  Unicef and Siamdutch  Don’t know  Other | 1077  286  80  194  215 | 58.2  15.4  4.3  10.5  11.6 | 1.0  0.77  0.99  0.94  0.89 | | 0.054  0.976  0.713  0.420 | | 0.59-1.00  0.62-1.58  0.69-1.29  0.66-1.19 |  |
| **RESPONDENT CHARACTERISTICS** | | | | | | | |  |
| **Respondent Years of Education***  None  1-6 years  7-9 years  10-12 years  13 or more years | | 330  293  732  353  144 | 17.8  15.8  39.5  19.1  7.8 | | 1.0  1.28  1.18  1.40  1.38 | 0.123  0.210  0.032  0.113 | 0.93-1.77  0.91-1.54  1.03-1.89  0.93-2.06 | |
| **Knows that mosquito transmits malaria***  No  Yes | | 261  1591 | 14.1  85.9 | | 1.0  1.42 | 0.009 | 1.09-1.84 | |

N = 1852

*Univariate significance level is p < 0.25

^OR, adjusted odds ratio; P-value for Wald statistic; CI, 95% confidence interval
